# Spontaneous Splenic Rupture and Rituximab-Induced Acute Thrombocytopenia in a Patient with High-Risk Mantle Cell Lymphoma

**DOI:** 10.1155/2019/2429098

**Published:** 2019-04-07

**Authors:** Jared Williams, Shingi Chiruka

**Affiliations:** Department of Haematology, Dunedin Hospital, 201 Great King Street, Dunedin 9016, New Zealand

## Abstract

Mantle cell lymphoma is a relatively rare type of mature B-cell non-Hodgkin's lymphoma with an incidence of approximately 8 cases per million persons per year. In patients with mantle cell lymphoma, there are rare case reports of the potentially life-threatening consequences of splenic rupture and rituximab-induced acute thrombocytopenia (RIAT) occurring separately, but there are no reports of these occurring in the same patient. Whilst rare, they are important to be aware of as early detection may prevent fatal outcomes.

## 1. Introduction

Spontaneous splenic rupture secondary to malignancy and RIAT are both rare complications with only a limited number of case reports in the literature. Literature review revealed over 800 cases of spontaneous splenic rupture with only 8 cases in mantle cell lymphoma. The leading causes of spontaneous splenic rupture were malignancy (30%), infections (27%), inflammatory (20%), and drug/treatment related (9%) [1]. 22 cases of RIAT were identified with 13 of these occurring in patients with mantle cell lymphoma. We report a case of high-risk mantle cell lymphoma (Mantle Cell Lymphoma International Prognostic Index Score 7.9) who developed these 2 rare complications.

## 2. Case Summary

A 53-year-old Caucasian female presented to her general practitioner with a 2-month history of increasing lymphadenopathy in her groin and neck alongside drenching night sweats, fatigue, and left upper quadrant abdominal pain but no subjective weight loss. Her medical history was significant for depression and prior transient thyrotoxicosis due to thyroiditis (negative thyroid antibodies), and she was taking paroxetine 20 mg daily. Fine needle aspiration was performed on a cervical lymph node suggestive of mantle cell lymphoma, which was subsequently confirmed by excision biopsy (CD5+, CD20+, cyclin D1+, CD23−, CD10−, CD3−, and t(11; 14) on FISH with Ki-67 of 50%). She was initially referred for outpatient assessment but admitted for inpatient workup because a rapidly increasing peripheral lymphocyte count was noted. On admission, multiple small volume lymphadenopathy was present in her cervical, axillary, and inguinal areas, and she had hepatosplenomegaly, with the spleen palpable 8 cm below the costophrenic margin and liver palpable 12 cm below the costophrenic margin. Significant blood results were as follows: total white blood cell count 76.8 × 10^9^/L, lymphocyte count 63.7 × 10^9^/L, haemoglobin 128 g/L, platelets 156 × 10^9^/L, neutrophils 10.5 × 10^9^/L, B_2_M 9.29 mg/L, LDH 704 U/L, uric acid 0.44 mmol/L, bilirubin 4 *µ*mol/L, ALP 211 U/L, GGT 104 U/L, ALT 19 U/L, and albumin 27 g/L with normal calcium, phosphate, and renal function. Hepatitis B antigen, hepatitis B surface antibody, and hepatitis B core antibody were negative. Hepatitis C antibody was negative. The morphology of the lymphocytes on blood film was consistent with mantle cell lymphoma cells, pleomorphic variant. Staging CT revealed a maximal liver span of 25 cm and spleen 19.8 cm with low-volume lymphadenopathy, the largest being 32 × 26 mm in the right inguinal area. Bone marrow confirmed marrow involvement, and the morphology of the cells were consistent with the pleomorphic subtype. She was commenced on the Nordic protocol and had her first dose of R-Maxi-CHOP (rituximab, cyclophosphamide, doxorubicin, vincristine, and prednisone) as an inpatient over 2 days. She had fevers during rituximab but no other significant infusion reactions. The evening after receiving CHOP, she developed abdominal pain with the haemoglobin dropping from 106 to 78 g/L and CT scan showed a large splenic rupture, with a subcapsular splenic haematoma and blood extending around the liver and in the paracolic gutter. There was no active bleeding on angiography. Her platelets had dropped from 154 to 50 × 10^9^/L. She remained haemodynamically stable. She was transfused 2 units of packed red blood cells and managed conservatively with no further decrease in haemoglobin. Routine pegfilgrastim (6 mg) had been previously charted and was given the following morning prior to medical review. She developed shortness of breath over the next 2 days, and chest X-ray revealed a significant left-sided pleural effusion which required thoracentesis. The effusion was an exudate with mantle lymphoma cells present. She was discharged from hospital a week later. Her 2^nd^ cycle was rituximab and cytarabine, and during this, she developed multiple infusion reactions to the rituximab and was only able to finish 14% of the rituximab as a result. Her platelet count dropped from 591 to 136 × 10^9^/L after the rituximab. Her lymphocyte count was normal after cycle 2 at 1.5 × 10^9^/L. 3^rd^ cycle of chemotherapy was delayed due to *Campylobacter* Gastroenteritis which was treated for 5 days with azithromycin. 11 days later following recovering from the infection, she presented for her 3^rd^ cycle of chemotherapy but her lymphocyte count had risen to 20.7 × 10^9^/L. Flow cytometry confirmed these were mantle cell lymphoma cells. Restaging CT showed stable disease compared with the original CT scan. Based on the increase in peripheral blood lymphocyte count, she was considered to have progressive disease. She was changed to salvage therapy with R-ICE (rituximab, ifosfamide, carboplatin, and etoposide). She again had recurrent infusion reactions with rituximab but this time developed significant acute thrombocytopenia following rituximab with platelet count falling from 197 to 22 × 10^9^/L within 24 hours. Her coagulation studies remained normal, and no other cause for the thrombocytopenia was found. This was presumed to be an acute toxicity of rituximab. No further rituximab was subsequently given due to concerns regarding the progressive fall in the postrituximab platelet count. Her platelet count recovered spontaneously without needing platelet transfusion. She tolerated the ICE well. Her lymphocytes fell to 2.5 × 10^9^/L then rose again to 5 × 10^9^/L. This was again thought to be progressive disease, and she was changed to DHAP (dexamethasone, cytarabine, and cisplatin). She tolerated this well, and lymphocytes decreased to 1.1 × 10^9^/L before rising to 7.2 × 10^9^/L 3 weeks later. DHAP was therefore ceased. At this stage, she had disease refractory to 3 lines of treatment and was considered for allogeneic stem cell transplantation. During workup for this, her disease was unable to be controlled and her management was changed to palliative and she passed away within 2 months.

## 3. Discussion

Splenic enlargement occurs in up to 40% of patients with mantle cell lymphoma but spontaneous splenic rupture is a rare phenomenon. Literature review has identified at least 800 cases of spontaneous splenic rupture, with 30% of these being related to malignancy [1]. There are 8 case reports of splenic rupture in mantle cell lymphoma [2–8]. In the majority of these, the mantle cell lymphoma was the blastoid variant and splenic rupture was the presenting complaint leading to the diagnosis, whereas in our patient, this occurred shortly after the 1^st^ cycle of chemotherapy. Risk factors generated from case reports include degree of splenic enlargement, older age, male sex, aggressiveness of underlying disease, fungal infection, growth factor usage, particularly granulocyte colony-stimulating factor (G-CSF), and induction chemotherapy [5]. Our patient's splenic rupture occurred closely following induction chemotherapy. Induction chemotherapy, when the disease burden is at its highest, in theory may cause tumor lysis with release of enzymatic content from the cells, resulting in splenic damage and rupture [5]. Our patient was also taking paroxetine, which can impair platelet function. After the rituximab, she became acutely thrombocytopenic (platelet count 50 × 10^9^/L); therefore, the paroxetine may have contributed to the bleeding. The pegylated G-CSF was given the day following the splenic rupture so was not contributory, but may have contributed to neutrophil activation exacerbating enzymatic digestion of the ruptured spleen. This had been charted alongside the chemotherapy and was routinely given the following morning prior to medical review. Management often involves splenectomy as the mortality in the case reports was high without surgical intervention. In the case reports of spontaneous splenic rupture in mantle Cell Lymphoma, 1 died at presentation and the majority of the others had some degree of haemodynamic instability and required urgent splenectomy. Our patient was haemodynamically stable, had no evidence of active bleeding, and therefore was managed conservatively without complication. Based on the limited number of mantle cell lymphoma case reports, it is hard to make recommendations for the management of spontaneous splenic rupture. However, splenectomy appears to be warranted, especially if there is any haemodynamic compromise.

Rituximab-induced acute thrombocytopenia (RIAT) is a rarely encountered side effect of rituximab. There are 22 case reports of this, and the majority of these have been in mantle cell lymphoma patients [9–25]. Omura et al. [9] listed the characteristics of these patients and noted the underlying diagnoses included mantle cell lymphoma (*n*=13) [10–18], hairy cell Leukemia (*n*=3) [11, 19, 20], follicular lymphoma (*n*=2) [9, 21], prolymphocytic Leukemia (*n*=1) [22], lymphoplasmacytic Lymphoma (*n*=1) [23], pre-B cell acute lymphoblastic leukemia (*n*=1) [24], and autoimmune haemolytic anaemia (*n*=1) [25]. From the available characteristics given in these case reports, all had bone marrow infiltration and splenomegaly similar to our patient. Many, but not all of the cases, had recurrent infusion reactions during rituximab doses similar to our patient. Looking back at our patient, she had acute thrombocytopenia following rituximab shortly after the first dose, but this was overlooked due to other factors including the splenic rupture after cycle 1, and after cycle 2, her platelet count was 591 × 10^9^ prerituximab masking the drop. The postrituximab percentage decrease in the platelet count was increasing with subsequent cycles (following cycle 1—68% decrease, cycle 2—77% decrease, and cycle 3—89% decrease). 5 of the 22 cases reports developed coagulopathy with the thrombocytopenia [9, 19–21, 24], suggesting consumption coagulopathy; however, our patient's coagulation studies remained normal. 12 cases attempted retrial of rituximab [9, 10, 12, 15–18, 23, 24] with 6 developing recurrence of thrombocytopenia [9, 10, 12, 15, 16, 24], making it difficult to decide clinically whether to continue it or not. It is likely that RIAT is underreported as it is not routine to monitor platelet counts after rituximab as most rituximab is given in the outpatient setting, and if the thrombocytopenia is mild-moderate, it may be overlooked.

The fact that these 2 seemingly rare events occurred in our patient may be a coincidence. From review of the literature, there are no case reports where these have occurred together. They are both rare events with limited case reports but both have a correlation with splenomegaly and mantle cell lymphoma.

## 4. Conclusion

This case report identified 2 rare events occurring in a patient with mantle cell lymphoma. As there are no other case reports of these occurring together, it is likely they are unrelated but highlight the importance of being aware of these potentially life-threatening complications.
